# Scalp psoriasis and Dermatology Life Quality Index: A retrospective study based on 12-year data from the Malaysian Psoriasis Registry

**DOI:** 10.51866/oa.146

**Published:** 2022-10-20

**Authors:** Wei Cheng Leong, Jyh Jong Tang

**Affiliations:** 1 MBBS (IMU), MRCP(UK), Adv M Derm (UKM), Department of Dermatology Hospital Raja Permaisuri Bainun Ipoh, Jalan Raja Ashman Shah, Ipoh, Perak, Malaysia. Email: leongweicheng@hotmail.com; 2 MD (UKM), MRCP(UK), Adv M Derm (UKM), Department of Dermatology Hospital Raja Permaisuri Bainun Ipoh, Jalan Raja Ashman Shah, Ipoh, Perak, Malaysia.

**Keywords:** Scalp psoriasis, Malaysia, Psoriasis

## Abstract

**Introduction:**

Psoriasis affects approximately 2–3% of the population worldwide, although the overall prevalence in Asia is <0.5%. Scalp psoriasis is a common initial presentation of psoriasis, which affects almost 80% of patients with psoriasis.

**Method:**

This retrospective descriptive study investigated 1,671 patients with psoriasis with scalp involvement registered with the Malaysian Psoriasis Registry (MPR) from January 2007 to December 2018.

**Results:**

A total of 21,859 patients with psoriasis were registered with the MPR during the study period; among them, scalp involvement was seen in 7.6% (n= 1,671). Female sex preponderance (61%) was observed in the majority of Malay patients (58.5%), followed by the Chinese (16.9%), Indian (17.1%) and other ethnic patients (7.5%). A positive family history of psoriasis was identified in 22.7% (n=380). Approximately 34.8% (n=581) and 11% (n=172) of the patients had nail changes and psoriatic arthropathy, respectively. The mainstay treatment modality was topical treatment (93.6%), followed by systemic therapy (10%) and phototherapy (0.5%). The comorbidities found among the patients with scalp psoriasis included hypertension (27.9%), obesity (26%), dyslipidaemia (21%), diabetes mellitus (18.4%), ischaemic heart disease (5.4%) and cerebrovascular disease (1.3%). Approximately 23% reported a Dermatology Life Quality Index (DLQI) of >10, which indicated moderate-to-severe impairment.

**Conclusion:**

The proportion of patients with psoriasis with scalp involvement in our study (7.6%) is much lower than previous reports. Scalp psoriasis markedly negatively impacts the DLQI.

## Introduction

Psoriasis is a common, chronic, immunologically mediated inflammatory disease with polygenic predisposition and is associated with triggering environmental factors.^1^ It affects approximately 2–3% of the population worldwide, although the overall prevalence in Asia is <0.5%.^2^ Scalp psoriasis is a common initial presentation of psoriasis, which has been reported to affect almost 80% of patients with psoriasis.^3^ It is characterised by sharply demarcated scaly lesions with silvery-white scale, which often advance beyond the hair border to the face or retro-auricular region.^4^ Scalp psoriasis can be psychologically and socially distressing.^5^ It poses a therapeutic challenge, as it is difficult to treat, although many treatment modalities are available. Herein, we describe the demographics, clinical characteristics, treatment given and quality of life among patients with scalp psoriasis in Malaysia using data obtained over a 12-year period from the Malaysian Psoriasis Registry (MPR).

## Methods

This retrospective descriptive study investigated 1,671 patients with psoriasis with scalp involvement who were registered with the MPR from January 2007 to December 2018. The demographic data, medical history, clinical findings, treatment modalities and Dermatology Life Quality Index (DLQI) were obtained for descriptive analyses. Published in 1994, the DLQI was the first dermatology-specific quality of life questionnaire. It consists of 10 questions concerning patients’ perception of the impact of skin diseases on different aspects of their health-related quality of life (HRQOL) over the last week. The domains include symptoms and feelings, daily activities, leisure, work and school, personal relationships and treatment. The total score ranges from 0 to 30, with higher scores indicating a greater impact on HRQOL.^6^ A DLQI of >10 indicates moderate-to-severe impairment of HRQOL. Descriptive statistics were presented as numbers and percentages for categorical variables. Means with standard deviations were used for normally distributed data and medians with interquartile ranges for non-normally distributed data. Collected data were tabulated using the Statistical Package for Social Sciences for Windows version 22.0 (SPSS, Chicago, IL, USA).

## Results

A total of 21,859 patients with psoriasis were registered with the MPR during the study period, of whom 7.6% (n= 1,671) showed scalp involvement. Female patients (n=1,019) accounted for the majority of the patients with scalp psoriasis (61%), yielding a male-to-female sex ratio of 1:1.56. In terms of ethnicity, most patients with scalp psoriasis were Malay (58.5%, n=977), followed by Chinese (16.9%, n=282), Indian (17.1%, n=286) and other ethnicities (7.5%, n=126). A positive family history of psoriasis was identified in 22.7% (n=380) of the patients. The mean age at onset was earlier in the women (29.16±16.78 years) than in the men (35.53±17.41 years). (**Table 1**)

**Table 1:** Sociodemographic characteristics of the patients with scalp involvement (n=1,671).

	n (%)
*Sex*	
Female	1019 (61.0)
Male	652 (39.0)
*Ethnicity*	
Malay	977 (58.5)
Chinese	282 (16.9)
Indian	286 (17.1)
Others	126 (7.5)
*Family history*	
Positive	380 (22.7)
Negative	1291 (77.3)
*Mean age at onset*	
Male	35.53+17.41
Female	29.16±16.78

**Figure 1:**
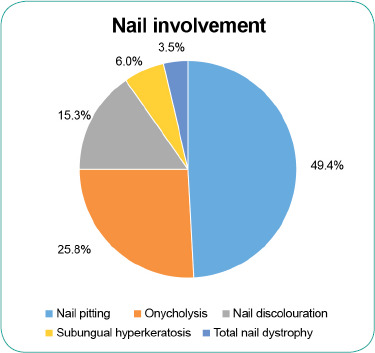
Nail involvement in the patients with scalp psoriasis

**Figure 2:**
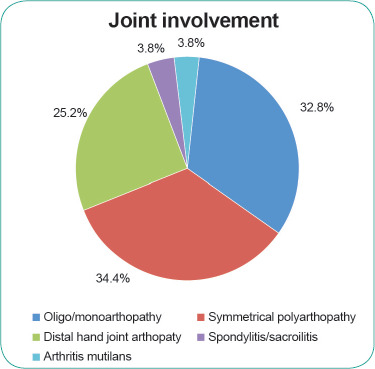
Joint involvement in the patients with scalp psoriasis

Nail changes were seen in 34.8% (n=581) of the patients, of which nail pitting was the most common finding (49.4%) (**Figure 1**). Arthropathy was seen in 11% (n=172) of the patients with scalp psoriasis. Symmetrical polyarthropathy was the predominant form observed (34.4%), followed by oligo/ monoarthropathy (32.8%) and distal hand joint arthropathy (25.2%); spondylitis/ sacroiliitis and arthritis mutilans each accounted for 3.8% of the scalp psoriasis cases associated with arthropathy. (**Figure 2**)

Regarding the treatment modalities for patients with psoriasis with scalp involvement, the majority (93.6%) (n=1,542) were using topical treatment. Topical corticosteroids were most commonly used (81.1%), followed by tar preparations (73.3%), keratolytics (46.2%), calcipotriol with betamethasone dipropionate (11.5%) and vitamin D analogues (11.2%). Phototherapy was prescribed for 0.5% (n=10) of the patients, of whom all received narrow band UVB. Systemic therapy was used by 10% (n=164) of the patients with scalp psoriasis in the MPR. Methotrexate was the most commonly prescribed form of systemic treatment (68.3%), followed by sulphasalazine (15.2%), acitretin (11.6%), cyclosporin (2.4%) and systemic corticosteroids (1.8%). Biologics accounted for only 4.3% of the systemic treatments used by the patients with scalp psoriasis.

Regarding comorbidities, hypertension was the most common type observed (27.9%), followed by obesity (26%), dyslipidaemia (21%), diabetes mellitus (18.4%), ischaemic heart disease (5.4%) and cerebrovascular accident (1.3%).

The DLQI was recorded in 39% (n=651) of the patients with scalp psoriasis in the MPR. Of these patients, approximately 23% reported a DLQI of >10, which indicated moderate-to-severe impairment of HRQOL. A DLQI of 0-1 (no effect on patient’s HRQOL) was obtained in 16.1%; a score of 2-5 (small effect on patient’s HRQOL) in 34.8%; a score of 6-10 (moderate effect on patient’s HRQOL) in 25.8%; a score of 11-20 (very large effect on patient’s HRQOL) in 20.4%; and a score of 21-30 (extremely large effect on patient’s HRQOL) in 2.9%.

## Discussion

### Demographics

Scalp involvement was seen in 7.6% (n=1,671) of all patients with psoriasis registered with the MPR (n=21,859) from 2007 to 2018. This proportion (7.6%) is significantly lower than previous findings.^7^ The frequency of scalp involvement in patients with psoriasis has been reported to range from 50% to 80%^8^; thus, the scalp represents the most commonly involved area of the body. Scalp psoriasis can be observed at the onset of disease or later. Scalp involvement can co-occur with any other type of psoriasis and at different phases of the disease (initial, intermittent or chronic).^9^ A 2017 nationwide survey of over 12,000 patients with psoriasis in China reported a mean age at onset of scalp disease of 30±14 years in men and 27±15 years in women,^10^ which is comparable to the mean age at onset of scalp psoriasis in our study (35.53±17.41 years in men; 29.16±16.78 years in women). The male-to-female sex ratio among the patients in our study was 1:1.56, showing a slightly higher prevalence among women, which was comparable to the ratio of 1:1.15 obtained by Egeberg et al.^7^ Our findings of a positive family history of scalp psoriasis among 22.7% of the patients is similar to those from a study conducted in China, which reported a positive family history among 23.1% of 12,0 patients with psoriasis.^10^

### Clinical features

The proportion of patients with nail changes in our cohort was lower than the 57.1% observed in the MPR from 2007 to 2016.^2^ Nail pitting was the most common form of nail change observed, which is similar to prior MPR findings.^2^ The prevalence of psoriatic arthropathy has been reported to range from 6% to 42% in patients with psoriasis.^11^ The cause of the wide variation may be attributed to the different screening strategies employed.^11^ The prevalence of psoriatic arthropathy in the patients with scalp psoriasis in the MPR was 11% compared with 13.7% in the MPR between 2007 and 2016.^2^ The most common pattern of psoriatic arthropathy in our study was symmetrical polyarthropathy (34.4%), followed by oligo/monoarthropathy (32.8%). This differs from MPR findings from 2007 to 2016, which showed oligo/monoarthropathy (37.3%) to be the predominant form, followed by symmetrical polyarthropathy (30.6%).^2^ In comparison, Kumar et al. showed the symmetrical polyarticular form to be the most common pattern (58%), followed by spondyloarthropathy (49%), asymmetric oligoarthritis (21%), isolated spondyloarthropathy (5%), predominant distal interphalangeal arthritis (3%) and arthritis mutilans (1%).^11^

The association between psoriatic arthropathy and other features of psoriasis is well known. The risk of developing psoriatic arthropathy is increased by 3.89 times among patients with scalp psoriasis compared with that among those without scalp psoriasis.^12^ Similarly, patients with psoriasis and nail dystrophy had a threefold higher risk of developing psoriatic arthritis than those without nail dystrophy.^12^

### Treatment

Psoriasis can be treated with topical therapy, phototherapy or systemic therapy. Topical therapy is recommended as the first-line treatment for all patients.^13^ Topical agents (93.6%) were also the mainstay treatment in our cohort study, with topical corticosteroids (81.1%) being the most common topical treatment prescribed. Wang et al. revealed topical corticosteroid therapy as the most common treatment for scalp psoriasis,^3^ which typically provides good control of the disease. This is followed by topical tar preparations, keratolytics, calcipotriol, vitamin D analogues, emollients and dithranol.^3,4^ The factors that may influence the choice of treatment include disease severity, patient preference, prior response and cost.^13^ Systemic treatments are usually provided to patients with scalp psoriasis with intractable diseases.^4^

### Comorbidities

The association between psoriasis and several systemic comorbidities is well known. Patients with psoriasis are 2.2 times more likely to have metabolic syndrome than the general population.^14^ The prevalence of comorbidities, including hypertension, dyslipidaemia, diabetes mellitus and ischaemic heart disease, among patients with scalp psoriasis in the MPR is higher than that among patients with psoriasis in China,^9^ for whom lower rates of hypertension (16.4%), dyslipidaemia (13.7%), diabetes mellitus (7.8%) and ischaemic heart disease (2.4%) have been reported. The findings of the comorbidities among the patients with scalp psoriasis in our study are similar to those from the MPR from 2007 to 2016, with hypertension (27.9%) representing the most common comorbidity reported, followed by obesity (26%), dyslipidaemia (21%), diabetes mellitus (18.4%), ischaemic heart disease (5.4%) and cerebrovascular accident (1.6%).^2^

### DLQI

Approximately 23% of our patients had a DLQI of >10, which indicated moderate-to-severe impairment of HRQOL. This rate is lower than that in the study conducted by Cakmur et al. in Turkey: 58% of the patients with scalp psoriasis reported a DLQI of >10.^15^ The most frequent and distressing symptoms of scalp psoriasis are itching and scaling.^5^ Scalp psoriasis is associated with significant impairments in the DLQI and negative impacts on patients’ self-confidence and social acceptance.^4,5,9^ This can be attributed to the visibility of the scalp region, which is considered a difficult-to-treat area, as scalp skin is relatively difficult to access, therefore reducing the efficacy of topical treatment.^16^ The significant number of patients with missing DLQI data (n=1,015) may limit the interpretation and generalisability of these findings.

### Limitations

The limitations of this study include the retrospective study design and the significant number of missing data, which might impact the accuracy of the results reported. Missing data could result from the lack of data collection from patients or inadequate documentation.

## Conclusion

This study describes the demographic characteristics, clinical features, treatment modalities and DLQI among patients with scalp psoriasis in Malaysia. Demographic studies on scalp psoriasis are limited worldwide. There is a need for more demographic studies on scalp psoriasis to help improve the care and treatment among patients.
